# Ultrasound-Assisted Osmotic Dehydration of Apples in Polyols and Dihydroxyacetone (DHA) Solutions

**DOI:** 10.3390/molecules24193429

**Published:** 2019-09-21

**Authors:** Joanna Cichowska, Dorota Witrowa-Rajchert, Lidia Stasiak-Różańska, Adam Figiel

**Affiliations:** 1 Department of Food Engineering and Process Management, Warsaw University of Life Sciences SGGW, Nowoursynowska 159c, 02-776 Warsaw, Poland; dorota_witrowa_rajchert@sggw.pl; 2 Department of Biotechnology, Microbiology and Food Evaluation, Warsaw University of Life Sciences WULS-SGGW, 159c Nowoursynowska St., 02-776 Warsaw, Poland; lidia_stasiak_rozanska@sggw.pl; 3 Institute of Agricultural Engineering, Wrocław University of Environmental and Life Sciences, 37/41 Chełmońskiego Street, 51 630 Wrocław, Poland; adam.figiel@upwr.edu.pl

**Keywords:** ultrasound, osmotic dehydration, polyols, sugar alcohols, water loss, apples

## Abstract

The aim of this work was to analyse the effect of ultrasound-assisted osmotic dehydration of apples v. Elise on mass transfer parameters, water activity, and colour changes. Ultrasound treatment was performed at a frequency of 21 kHz with a temperature of 40 °C for 30–180 min using four osmotic solutions: 30% concentrated syrups of erythritol, xylitol, maltitol, and dihydroxyacetone (DHA). The efficiency of the used solutes from the polyol groups was compared to reference dehydration in 50% concentrated sucrose solution. Peleg’s model was used to fit experimental data. Erythritol, xylitol, and DHA solutions showed similar efficiency to sucrose and good water removal properties in compared values of true water loss. The application of ultrasound by two methods was in most cases unnoticeable and weaker than was expected. On the other hand, sonication by the continuous method allowed for a significant reduction in water activity in apple tissue in all tested solutions.

## 1. Introduction

Osmotic dehydration (OD) is a simple technique for removal of water from fruits and vegetables, although a more correct term is “osmotic dewatering” since the final product still has a high moisture content. However, the amount of water remaining in the material does not ensure its stability, as water activity is generally higher than 0.9 [1]. This partial dehydration is performed by immersion of the fruit or vegetable material in a concentrated aqueous solution, where there are two major simultaneous countercurrent flows: one flow of solutes from the solution into the food matrix and another from the food into the osmotic solution (principally water) [2]. OD is usually used as a pre-treatment before drying and preserves texture and colour [1]. The relevance of osmotic dehydration is chiefly related to the improvement of some nutritional, functional, and organoleptic properties of the product [3]. Recently, different substances such as sweeteners or sweetness enhancer were proposed as an alternative to the use of sucrose. One of them was steviol glycoside, the additive which was used in OD [4]. Researchers also focused on solutes from the polyol groups, such as xylitol, erythritol, and maltitol [5,6,7]. The use of sugar alcohol during pre-treatment can reduce sugar content in the product, resulting in the reduction of calories [8].

Ultrasound is an example of a new form of technology, and its application in food processing are numerous and include among others pre-treatment in extractions, freezing, drying, defoaming, cleaning, depolymerization, disaggregation, and inactivation of microorganisms [9]. Primarily, ultrasonic waves with high power at low frequency (20–100 kHz) are applied at a minimal temperature to stimulate a rapid series of alternative expansions and compressions, resulting in the removal of moisture and providing a sponge-like effect [3]. The stress can generate micro-cracks in the internal structure, producing micro-channels that facilitate moisture transport. Moreover, high-intensity ultrasound can produce cavitation in the liquid fraction and the asymmetric implosion of cavitation bubbles near to the solid surface, leading to a partial release of some water bounded to the solid structure. All these mechanical effects result in a reduction in the internal resistance to mass transport and, therefore, an increase in the internal diffusion of water [10]. Ultrasound also induces changes on the cell structure, but in contrast to osmotic dehydration no cell breakdown is observed, and the increase in diffusivity is attained by the formation of microscopic channels in the cell structure, which also offer lower resistance to diffusion of water, pigments and soluble solids [11]. The beneficial use of sound is realized through its chemical, mechanical, or physical effects on the process or product [9]. The application of continuous high-frequency ultrasound enhances the mass transfer rate during osmo-concentration. Ultrasound in combination with high sugar concentration speeds up the rate of water withdrawal from the tissue and may significantly reduce the osmo-dehydration time [3,12]. Ultrasound application may also change the viscosity and surface tension, and deform porous solid materials. During ultrasound application, no increase of intercellular spaces has been reported in the literature [11]. Nowacka et al. [13] investigated the utilization of ultrasound as a mass transfer-enhancing method prior to drying of apple tissue. The ultrasound treatment caused a reduction of the drying time by 31–40% in comparison to untreated tissue.

The aim of this study was to investigate the effect of the application of ultrasound (using two methods—continuous and with intervals) during osmotic dehydration in polyols solutions on the mass transfer and water removal from apple tissue. The influences of ultrasound treatment on water activity and colour changes during the process were analysed as well.

## 2. Results and Discussion

### 2.1. Water Content (WC)

The raw apple tissue was characterized by a water content (WC) of 5.69 ± 0.25 g H_2_O/g dry matter (Table 1). With the increase of time followed loss of WC in fruit. The lowest values were observed after OD in the reference dehydration in sucrose solution; at the end of the process, the WC was reduced to 1.79 ± 0.2 g H_2_O/g d.m. A 50% reduction in the WC of raw apple was obtained after 60 min in the case where sucrose used as osmotic agent (except OD with interval sonication), and after 120 min and 150 min in the case of erythritol and xylitol, respectively (Table 1). Simal et al. [14] dehydrated apple cubes in 70°. Brix sucrose solution. They reported a 50% reduction in water content after 150, 105, 90, and 75 min at 40, 50, 60, and 70 °C, respectively. Application of ultrasound allowed for this time to be reduced to 105, 90, 60, and 50 min, respectively.

Osmotic dehydration in maltitol and DHA solutions resulted in a lower level of reduction water content—at the end of the process values of water content were higher than 3 g H_2_O/g d.m., while in the case of sucrose, water content was at the level of about 2 g H_2_O/g d.m. (Table 1). In almost all cases the highest values of water content were noted for OD with the application of ultrasound by the interval method. However, statistical analysis did not show any differences between the values of WC achieved in the case of sonication (continuous method), compared to after OD without treatment (Table 2). Moreover, the application of ultrasound by the interval method resulted in an increase of the observed values. This phenomenon is most noticeable in the case of OD in DHA solution (Table 1). Similar results were reported by Nowacka et al. [13], who stated that with the increase in the applied treatment time of ultrasound (from 10 to 30 min) there was a loss of dry matter in fruit and the changes were significant compared to untreated samples. Additionally, the change of ultrasound frequency resulted in a dry matter decrease. Namely, the application of 21 kHz, which was also used in the present research, led to higher changes than using 35 kHz [15]. Mierzwa and Kowalski [16] reported that the most effective period of dehydration took place in the first 30 min of the process, regardless of the type of osmotic agent (fructose/sorbitol) and the variant of the process (with or without sonication).

Phisut et al. [17] dehydrated cantaloupe by two methods: slow and fast osmotic dehydration (SOD and FOD, respectively). In FOD, the cantaloupe slices were immersed continuously in 50° Brix sucrose solution for 24 h, but in SOD, the cantaloupe slices were first immersed in 30° Brix sucrose solution for 24 h and the slices were then transferred to a 40° Brix sucrose solution for 24 h. After that, the slices were transferred to a 50° Brix sucrose solution for another 24 h. No difference in moisture content was found between sample produced by FOD and SOD (*p*-value >0.05). The same results were achieved by Fei et al. [18], who osmo-dehydrated button mushrooms. The water contents in ultrasound-assisted osmo-dehydrated samples and OD samples showed no significant differences, but they were lower than in control samples. 

Osmotic dehydration supported in ultrasonic pre-treatment can result in different behaviour of the fruit: gaining or losing water during pre-treatment. For example bananas, sapotas, papayas, and jenipapos gained water during ultrasound treatment. However, melons and pineapples lost a small amount of water during pretreatment [11]. Simal et al. [14] reported that apple cubes subjected to sucrose and treated by ultrasound, dewatered faster than non-treated samples. Water and solute transport rates were significantly higher in sonicated samples in comparison with those not sonicated during osmotic dehydration. The higher water content after sonication in the present research (Table 1, compared to osmo-treated samples) could be explained by the higher the loss of soluble solids from the tissue.

### 2.2. Water Loss (WL)

**Figure 1:**
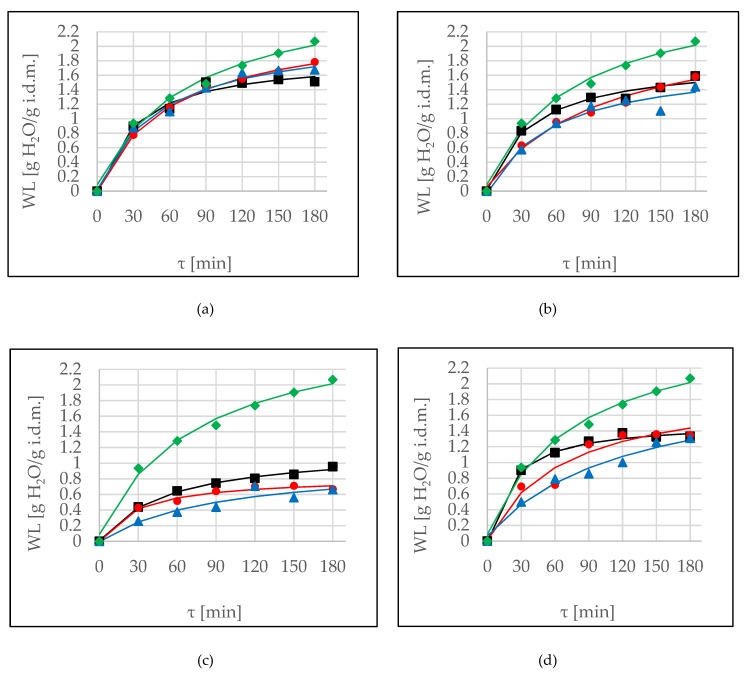
Water loss (WL) kinetics at several conditions (OD (■), OD+US (●), OD+US_i_ (▲)) at 40 °C, in different solutions: (**a**) erythritol, (**b**) xylitol, (**c**) maltitol, (**d**) DHA. Lines are the Peleg’s model. The green line (♦) is the kinetic reference (sucrose).

Water loss is a parameter which allows for the evaluation of the effectiveness of osmotic dehydration. In the case of OD, in sucrose solution the achieved values were the highest (Figure 1, green lines). This observation remained in agreement with research by Nowacka et al. [4], where the highest water loss was noticed for samples treated in sucrose as compared to trehalose and with the addition of steviol glycoside.

Among the tested solutions, the most comparable WL values were noted when erythritol was used as the osmotic agent (Figure 1a). At the end of the process, observed values were in the range of 1.6–1.8 g H_2_O/g initial dry matter. This is because of erythritol having a lower molecular weight than other osmotic agents (xylitol, sucrose, maltitol) do. The use of hypertonic solution, which has low molecular weight, increased the phenomenon of water loss [6]. In this case there was no significant influence of sonication (*p*-value = 0.276). The second most effective osmotic agent, which can be used as an alternative to sucrose, proved to be xylitol (Figure 1b). However, application of ultrasound (both methods) resulted in a decrease of WL values. A similar situation was observed in the case of OD in maltitol solution (Figure 1c). Moreover, due to low values of WL, this solute at a tested concentration of 30% was considered to be ineffective. However, Phisut et al. [8] in the case of cantaloupe noted higher water loss and a solid gain in maltitol-treated sample compared to a sucrose-treated sample when a 50% concentrated solution was used.

The opposite results were reported by Mierzwa and Kowalski [16] during ultrasound-assisted OD in fructose solution (40% concentration), achieving noticeably higher values (compared to untreated samples). Also, Nowacka et al. [4] reported that ultrasound pre-treatment led to a significant increase in water loss during OD of cranberries. After 90 min, papaya subjected to ultrasound resulted in the largest loss of water (11.92%), while at shorter ultrasound treatment time the water loss was lower. However, between OD and ultrasound-assisted OD samples no statistical differences were found [19]. Simal et al. [14] reported the applicability of sonication to osmotic dehydration of porous fruit such as apple cubes and showed that the rates of mass transfer increase with the use of ultrasound in comparison with the osmotic process carried out under dynamic conditions involving 50 RPM of agitation. 

Continuous sonication method during OD in DHA solution (Figure 1d) did not influence significantly on achieved values, while the interval method resulted in weakness of the phenomenon. In almost all cases (except point 180 min for xylitol) the processing time had a significant influence on observed values up to 120 min. The further prolonging of the process did not affect the parameter increase. Multifactor ANOVA confirmed the significant influence of all of the factors: time, type of osmotic agent, and method of application on achieved values (Table 3).

Modeling of WL kinetics by Peleg’s model was efficient in all of the cases. High R^2^ values, low values of the root mean square error (RMSE), and coefficient of residual variation (CRV) <20% means that this model can be used for prediction of WL (Table 4). During the application of ultrasound in almost all cases, an increase of the *k*_1_ parameter was observed as well as a decrease of the *k*_2_ parameter. This means that an initial mass transfer rate at the beginning of the process was weaker under sonication. However, the *k*_2_ defined the equilibrium value of WL (and soluble solids) [6], and consequently the water removal was higher under pre-treatment. Observed values of *k*_2_ parameter were higher compared to OD in sucrose, on the other hand, the value of *k*_1_ in control OD was low, which means high dehydration rate at the very beginning of the process (Table 4).

### 2.3. Solid Gain (SG)

**Figure 2:**
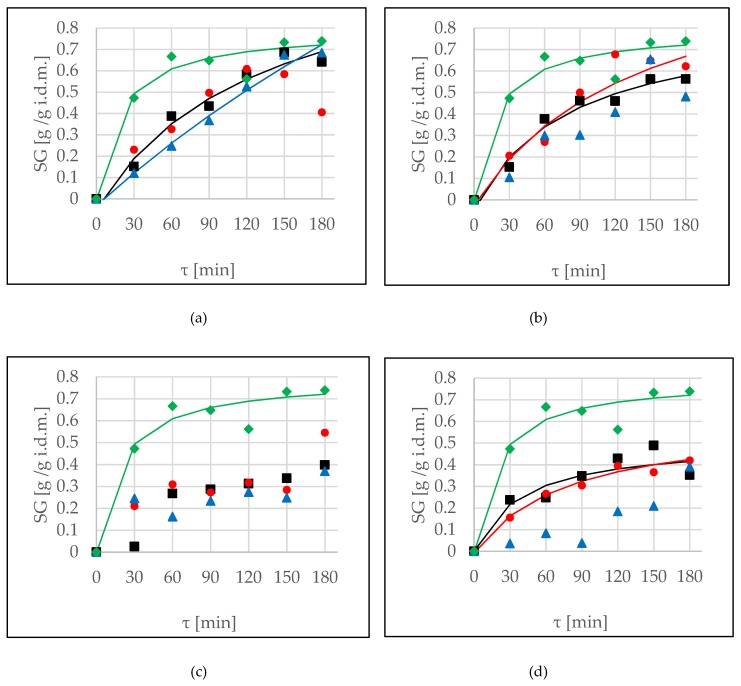
Solid gain (SG) kinetics at several conditions, (OD (■), OD+US (●), OD+USi (▲)) at 40 °C, in different solutions: (**a**) erythritol, (**b**) xylitol, (**c**) maltitol, (**d**) DHA. Lines represent the Peleg’s model. The green line (♦) is the kinetic reference (sucrose).

During OD, the phenomenon of solid gain was also observed. The main aim of this research was to remove water (as much as possible) from the apple tissue, not to enrich it in additional compounds. Figure 2 shows kinetics of SG using different osmotic agents during osmotic dehydration and ultrasound application by two methods. Similarly to the WL parameter discussed above, sonication did not have significance in achieved values of SG in the case of OD in erythritol (*p*-value 0.182) (Figure 2a). The interval method of US application did not cause significant differences compared to values without treatment in the cases of use of xylitol and maltitol solutions (Figure 2b,c). In these cases, sonication by continuous method resulted in an increase of SG values. The kinetics of OD in xylitol solution (Figure 2b) were similar to the kinetics of 50% concentrated glycerol at 25 °C [20]. Moreover, the kinetics of OD together with sonication by the interval method were similar to those of OD in 60% concentrated glycerol at 35 °C [20]. A different behaviour of apple tissue was observed during OD in DHA solution (Figure 2d). The interval method significantly decreased values of SG, whereas the values achieved during the continuous method of ultrasound application were classified into one homogenous group with those which were obtained after OD without sonication. Also, Fei et al. [18] reported that the solid gain in the OD samples was significantly higher (*p* < 0.05) than that in the ultrasound-assisted OD samples. This result could be attributed to the over twice shorter treatment time for OD samples supported with ultrasound.

Over the 2 hours of the process there was further enrichment of the tissue, while WL was mainly observed up to this time (Table 5). The smallest solid uptake was noticed in the cases of DHA and maltitol solutions, whereas the biggest was observed when erythritol and xylitol were used as osmotic agents (no statistically significant differences). Ambiguous results of the influence of sonication were also obtained by Mierzwa and Kowalski [16]: in the case of fructose, application of US resulted in higher values of WL; however, in the case of sorbitol, US-treatment resulted in smaller values. Mieszczakowska-Frąc et al. [21] reported that during application of ultrasound in a water medium was observed an increase of water content and substantial loses of soluble solids, whereas during sonication in sucrose solution a significant increase of WL and SG values was noted. These observations remained in consensus with results obtained by Fernandes et al. [22] in the case of melon. On the other hand, during OD of cranberries, using sucrose and trehalose as osmotic agents, sonication did not promote any differences for solid gain, while it caused a significant decrease in samples with steviol glycoside [4]. 

Using of the Peleg’s model to predict SG values was impossible in the case of maltitol (Figure 2c) due to CRV values higher than 20% and high values of the indicator of root mean squared error (RMSE) (Table 6). The same problem was reported in the previous research by Cichowska et al. [5] and in this case a better fit was found using the Kelvin–Voigt model. A similar situation was found in the case of presentation of SG kinetics for interval applications using xylitol and DHA solutions, as well as the continuous method in erythritol solution. Using the sonication resulted in an increase of k_1_ values (meaning that initial mass transfer was reduced) and decreased values of k_2_ parameters (Table 6). The lowest values of k_2_ parameter were noted in the case of OD in erythritol solution, in an agreement with the greatest water loss using this osmotic agent (Figure 1a).

### 2.4. True Water Loss (WL_T_)

**Figure 3:**
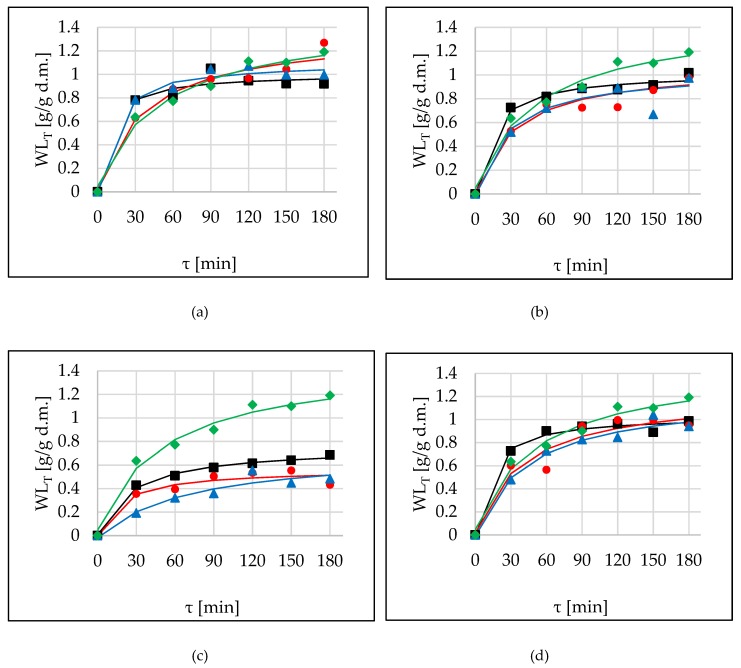
True water loss (WL_T_) kinetics in several conditions (OD (■), OD+US (●), OD+USi (▲)) at 40 °C in different solutions: (**a**) erythritol, (**b**) xylitol, (**c**) maltitol, (**d**) DHA. Lines are the Peleg’s model. The green line (♦) is the kinetic reference (sucrose).

WL_T_, a new parameter which was proposed by Cichowska et al. [5], describes real water loss, including actual solid uptake during OD. When comparing values of WL_T_ for different osmotic agents, excellent efficiency (similar to that of sucrose) and good water removal properties were found for erythritol, xylitol, and DHA solutions (Figure 3a,b,d). This behaviour of osmotic agents results from the high osmotic pressure of these substances, which were calculated by Cichowska et al. [5,6]. At the end of the process values of WL_T_ were in the range of approximately 0.9–1.2 g/g d.m. However, using 30% concentrated maltitol as an osmotic agent, achieved values were smaller (0.5–0.7 g/g d.m.) (Figure 3c). No statistical differences were observed between sonication and OD without US-treatment in the case of erythritol (*p*-value = 0.165) (Figure 3a). In other cases, application of ultrasound resulted in a decrease of WL_T_ values. Statistical analysis showed that time had a significant impact during 120 min of the process, so prolonging for a longer time is unfounded (Table 7). Generally, sonication by continuous method did not give better results compared to OD without US-treatment (no significant differences). Moreover, the interval method of ultrasound. 

The goodness of fit experimental data to Peleg’s model was effective in all tested solutions, as evidenced by high R^2^ values, low RMSE, and low χ2 values (Table 8). Similar to modelling WL and SG parameters, application of ultrasound resulted in an increase of *k*_1_ and decrease of *k*_2_ parameters in the cases of OD in erythritol, xylitol, and DHA solutions. The opposite situation took place using maltitol as an osmotic agent. The best water removal rate was characterized by sucrose (the lowest *k*_2_ parameter).

### 2.5. The Cichowska et al. Ratio

**Figure 4:**
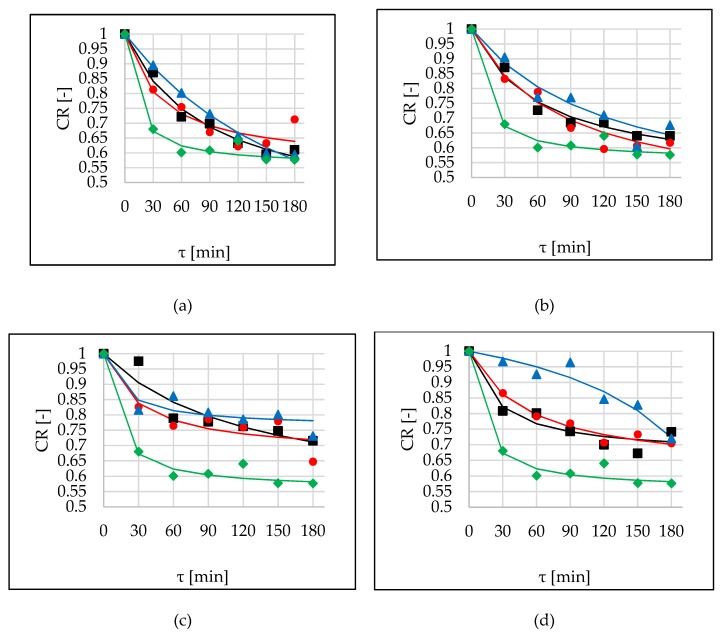
The Cichowska et al. ratio (CR) kinetics in several conditions (OD (■), OD+US (●), OD+USi (▲)) at 40 °C, in different solutions: (**a**) erythritol, (**b**) xylitol, (**c**) maltitol, (**d**) DHA. Lines are the Peleg’s model. The green line (♦) is the kinetic reference (sucrose).

The ratio of WL_T_/WL was defined as the Cichowska et al. ratio (CR) [5]. The lowest values of this parameter in the case of sucrose indicate that during OD solid uptake was considerable, with simultaneous major water loss (Figure 4, green lines). The opposite situation could be seen in the cases where both WL and SG were inconsiderable, as in the case of maltitol (Figure 4c), or when WL was significant and at the same time SG was slight, as observed during OD in DHA solution (Figure 4d). This ambiguity means that this parameter should be analysed together with the two parameters WL and SG. For this research, the second situation was desirable. After 120 min of OD, which was considered the most optimal for ending of the process, the highest values of CR were noted using DHA as an osmotic agent. The CR values were in the range of 0.77-0.87, and slightly lower in the cases of xylitol and erythritol: 0.7–0.65 and 0.65, respectively (Figure 4a,b). Sonication did not have any influence on CR values in the case of erythritol (*p*-value 0.105) (Figure 4a). Statistical analysis showed that higher values of this parameter were achieved when interval sonication was applied (Table 9).

Peleg’s model also can be used for the prediction of CR. Values of CRV are lower (Table 10) compared to parameters discussed above. Influence of sonication on model parameters in the case of erythritol was ambiguous. Similar to modelling of WL and WL_T_, application of ultrasound resulted in an increase of *k*_1_ and decrease of *k*_2_ parameters in the cases of xylitol and DHA solutions, and opposite in the case of maltitol. The most effective behaviour (the highest water removal and small solid uptake) showed DHA hypertonic solution, which was proved by high *k*_1_ and small *k*_2_ parameters.

### 2.6. Water Activity

**Figure 5:**
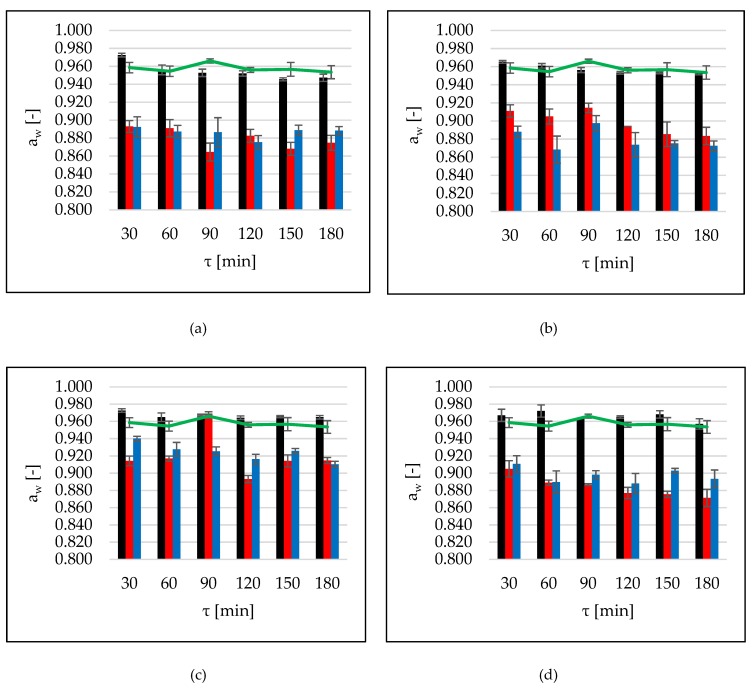
Water activity, a_w_, at several conditions (OD (black bars), OD+US (red bars), OD+USi (blue bars)) at 40 °C, using different solutions: (**a**) erythritol, (**b**) xylitol, (**c**) maltitol, (**d**) DHA. The green lines are values for the reference (sucrose).

Raw apple tissue was characterized by water activity of 0.967. The use of sugar alcohols could reduce a_w_ in the product. Hydroxyl groups of polyols can form hydrogen bonds with water, resulting in the increment of bound water in osmo-dried fruit [8]. With increment of time, the a_w_ values decreased during OD in erythritol and xylitol solutions (Figure 5a,b, black bars). In other cases (maltitol, DHA, sucrose), a_w_ remained at a similar level during all the process of OD without US treatment (Figure 5c,d, black bars). Application of ultrasound by continuous method allowed to decrease this parameter below the value of 0.880 after 90 min of OD in erythritol and after 120 min using DHA solutions. The interval method of application gave better results only in the case of xylitol used as osmotic agent. Values, compared with continuous method of sonication, were significantly lower. The lowest reduction of water activity was observed during OD in maltitol solution. It was related to the small osmotic pressure at the tested 30% concentration [5]. There were no significant differences between values achieved during OD in erythritol, xylitol, and sucrose solutions, as well as between xylitol, sucrose, and DHA used as osmotic solutes (Table 11). Decrease of a_w_ took place during 120 min of the process, which confirmed earlier observations. Significant reduction of this parameter is very desirable by producers because water activity determines food microbiological safety. It is worth mentioning that values of a_w_ significant decreased under the influence of sonication. Simultaneously, no significant influences of ultrasound on water content in the tissue were observed, and there were unnoticeable differences in WL (comparing values after sonication and without US-treatment). This could indicate the changes in the degree of water binding in the cell. It is possible that under influence of ultrasound reorganization of water molecules occurred, and thus they became less available. This hypothesis should be verified in the future research, using the method with nuclear magnetic resonance (NMR). 

Nowacka et al. [4], after 30 min of sonication and 72 h of OD in sucrose solution, achieved a_w_ values of about 0.867. Phisut et al. [17] studied the influence of fast osmotic dehydration (FOD) and slow osmotic dehydration (SOD) in sucrose solution on the chemical, physical, and sensory properties of osmo-dried cantaloupe. They observed that the SOD-treated cantaloupe sample showed lower water activity (0.69) as compared to the FOD-treated samples (0.72). These findings may be due to the higher sugar content of the SOD-treated sample, which encouraged the interaction of sugar and water molecules via the hydrogen bond. The same authors in another study [8] investigated the effect of osmotic dehydration in various solutions (sucrose, maltitol, sorbitol, and invert sugar) on cantaloupe tissue. They observed that sugar alcohols (sorbitol and maltitol) and invert sugar can reduce a_w_ in the osmo-dried product.

### 2.7. Colour Changes

Colour is the one of important discriminants of the main quality attributes that influence the product acceptance by the consumer [23]. The browning index (BI) represents the purity of the brown colour. Table 12 shows changes of this parameter during OD in different solutions. The lightest-coloured tissue was found in samples which were dipped into a hypertonic solution with sucrose (Table 12). Statistical analysis revealed that the time of osmotic treatment had no significant influence on BI values (Table 13). Moreover, the highest values of the BI parameter were noted during OD with interval sonication.

**Figure 6:**
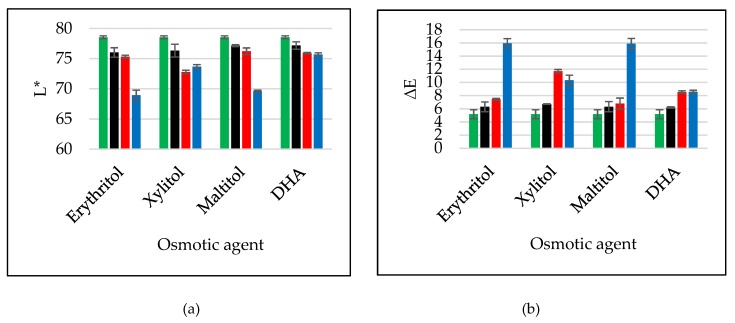
Colour changes after 120 min OD of apples: values of parameter L* (**a**), values of absolute colour difference (**b**) in several conditions (OD (black bars), OD+US (red bars), OD+USi (blue bars)) at 40 °C using different solutions. The green bars are values for reference (sucrose).

Figure 6 shows changes in the colour of the apple tissue after 120 min (the most optimal time for ending of the process). Values of the L* parameter were on a similar level after OD in polyols and DHA solutions. However, these values were slightly lower compared to sucrose (green bars) (Figure 6a). Sonication continuous method resulted in a decrease in values of L* about 1 unit in the cases of erythritol, maltitol and DHA solutions. When xylitol was used as an osmotic agent, the decrease equalled 3 units. Intervals application of ultrasound was insignificant on achieved values of L* and ∆E during OD in DHA solution. However, interval application of ultrasound resulted in the major darkness of the apple tissue, and consequently, high values of absolute colour differences in the cases of erythritol and maltitol (Figure 6ab). Opposite situation took place during OD in xylitol. More lightness of the surface and smaller ∆E compared to the second method were observed. Absolute colour differences after 120 min were on the similar level during OD in all tested hypertonic solutions (Figure 6b black bars).

L* values were higher in osmosed mangoes than in untreated samples; however, the colour of guava after ultrasound application became darker [24]. Ultrasound application has been showed to be unable to totally deactivate browning enzymes, such as polyphenoloxidase (PPO) and peroxidase (POD) [25]. The partial deactivation of these enzymes may lead to a certain degree of loss of lightness and the formation of brown compounds due to enzymatic browning [24]. 

More information about colour changes could show absolute colour differences. After osmotic dehydration without US-treatment small changes of ∆E were observed in the range of 4.8-7.5 (Table 14). Sonication by continuous method resulted in decrease of ∆E values in the case of control dehydration, but the interval method caused an increase of these values. Both methods of ultrasound application resulted in more changes observed in samples after OD in the case of other tested solutions. Moreover, the interval method brought two or three times more changes. This is in agreement with the parameter discussed above. The smallest changes in colour were observed when sucrose and DHA were used as the osmotic agent. Additionally, the processing time had no influence on observed changes (Table 15).

## 3. Materials and Methods

### 3.1. Sample Preparation

Fresh apples of the Elise variety were collected from the Experimental Fields (orchards) of the Faculty of Horticulture and Landscape Architecture (Warsaw University of Life Sciences). The fruits were stored at 4 ± 1 °C and relative humidity of 85–90% in a refrigerator until use. Before the experiment, the apples were washed, stoned, and cut into 5-mm-thick slices, and then each slice was cut into four pieces.

### 3.2. Pre-Treatment Procedure

In this procedure, a sample of 20 g ± 2 g was placed in a beaker into syrups in the ratio of 1:4 (fruit:solution) [4,26] in order to avoid significant changes in the solution concentration. Osmotic solutions were prepared with selected substances from the polyol group: erythritol, xylitol, and maltitol (Brenntag, Kędzierzyn-Koźle, Poland) as well as dihydroxyacetone (DHA) (Merck, Germany) dissolved in distilled water. Then, the beakers with samples immersed in osmotic solutions were positioned in an ultrasonic bath MKD-3 (MKD Ultrasonics, Stary Konik, Poland, internal dimensions: 240 × 140 × 110 mm). Two experimental repeats were carried out simultaneously. The temperature of the water bath was constant (40 °C). During sonication in OD solutions, significant temperature changes were not observed (±1 °C). The pre-treatment was conducted in the range from 0.5 to 3 hours by two methods: continuous and with 30-min intervals. The used frequency was 21 kHz and the total power generated by sonotrodes was 320 W, which corresponded to the ultrasound intensity of 8 W per gram of material. Afterwards, samples were removed from the osmotic solution, blotted with absorbent paper to remove osmotic liquid from their surface and were weighed.

### 3.3. Mathematical Modelling

Mass transfer parameters: WC, WL, and SG were determined according to Cichowska et al. [6] and WL_T_, CR according to equations by Cichowska et al. [5]. Fitting of the mathematical model (Peleg:$Y=Y_{0}\pm \frac{\tau }{\left(k_{1}+k_{2}\tau \right)}$) to the experimental points was done using Table Curve 2D version 5.01 (SYSTAT Software Inc., Chicago, IL, USA) [5]. The determination coefficient (R2), the reduced chi-squared statistic (χ2), the root mean square error (RMSE), and the coefficient of residual variation (CRV) were used to evaluate the goodness of fit of the model.

### 3.4. Water Activity

Water activity was determined using the AquaLab CX-2 (Decagon Devices Inc., USA) apparatus in accordance with the manufacturer’s instructions. The temperature of water activity determination was constant (25 °C). Each measurement was conducted in four repetitions.

### 3.5. Colour Measurement

Colour analysis of the surface of the osmo-dehydrated apple was determined with the use of Minolta Chroma Meter CR-200 (Minolta Corp., Osaka, Japan). The measurement conditions were: D65 standard illuminate, 2° Standard Observer, measurement diameter: 30 mm. The results were presented using the directly measured parameters: L* (lightness/darkness), a* (red/green), b* (yellow/blue). The measurements were made in 5 repetitions for every sample; the mean values were reported. The total colour differences (ΔE – Equation (1)) were calculated according to the following formula:

(1) $$\Delta E=\sqrt{{\left(\Delta L^{*}\right)}^{2}+{\left(\Delta a^{*}\right)}^{2}+{\left(\Delta b^{*}\right)}^{2}}$$

where ΔL*, Δa*, Δb* represent the change of L*, a* and b* parameters, respectively, between raw material and samples after treatment. 

The browning index (BI, Equations (2) and (3)) was calculated according to [27]:

(2) $$BI=\frac{100\times \left(X-0.31\right)}{0.172}$$

(3) $$X=\frac{a^{*}+\left(1.75\times L^{*}\right)}{\left(5.645\times L^{*}\right)+a^{*}-\left(3.012\times b^{*}\right)}$$

The browning index (BI) represents the purity of the brown colour and was calculated for each sample separately.

### 3.6. Statistical Analysis

The statistical software Statgraphics Plus ver. 5.1 (StatPoint) and Excel 2016 (Microsoft) were used for data analysis. The influence of pre-treatment (duration of the process, type of osmotic solution, method of ultrasound) on dependent variables: (WC, WL, SG, WL_T_, CR, a_w_ and colour changes) was evaluated by means of a multifactorial analysis of variance (ANOVA) at a significance level α = 0.05. In the case of significant associations, post-hoc Tukey’s test was performed.

## 4. Conclusions

The expected positive effect of ultrasound application on mass transfer intensification during osmotic dehydration turned out to be unnoticeable in the case of WC, WL, and SG parameters. Erythritol, xylitol, and DHA solutions at a 30% concentration showed similar efficiency to sucrose and good water removal properties based on values of true water loss (WL_T_) during osmotic dehydration. Sonication resulted in a decrease of WL_T_ parameter values; only in the case of erythritol were no statistically significant differences observed. Maltitol at the tested concentration was ineffective. Peleg’s model could be used for prediction of observed values for almost all parameters, except few cases of solid gain. Application of ultrasound by continuous method allowed us to significantly reduce water activity in apple tissue in all tested solutions and achieved small colour changes, using sucrose as an osmotic agent. The use of the interval method was unfounded because of too high changes in absolute colour changes and weakness of the phenomenon of water loss.

## Figures and Tables

**Table 1:** Water content (g H_2_O/g dry matter) in apples during OD in different solutions. OD: osmotic dehydration, US: continuous sonication, US_i_: interval sonication; DHA: dihydroxyacetone.

Time [min]	Solution/Application	Sucrose	Erythritol	Xylitol	Maltitol	DHA
**0**		5.69 ± 0.2				
**30**	OD	3.23 ± 0.2	4.16 ± 0.2	4.22 ± 0.4	5.11 ± 0.2	3.86 ± 0.1
	OD+US	3.52 ± 0.2	3.99 ± 0.1	4.20 ± 0.3	4.34 ± 0.1	4.31 ± 0.1
	OD+US_i_	3.82 ± 0.2	4.30 ± 0.3	4.62 ± 0.2	4.44 ± 0.2	5.01 ± 0.3
**60**	OD	2.64 ± 0.1	3.27 ± 0.0	3.31 ± 0.1	3.97 ± 0.1	3.65 ± 0.1
	OD+US	2.54 ± 0.2	3.41 ± 0.1	3.72 ± 0.2	3.95 ± 0.2	3.92 ± 0.2
	OD+US_i_	3.09 ± 0.5	3.67 ± 0.1	3.66 ± 0.2	4.57 ± 0.2	4.53 ± 0.3
**90**	OD	2.55 ± 0.2	2.91 ± 0.1	3.00 ± 0.1	3.83 ± 0.1	3.28 ± 0.2
	OD+US	2.96 ± 0.1	2.84 ± 0.2	3.06 ± 0.1	3.96 ± 0.1	3.42 ± 0.3
	OD+US_i_	2.81 ± 0.2	3.11 ± 0.1	3.47 ± 0.2	4.23 ± 0.2	4.65 ± 0.1
**120**	OD	2.52 ± 0.0	2.64 ± 0.0	3.02 ± 0.0	3.71 ± 0.1	3.01 ± 0.0
	OD+US	2.32 ± 0.1	2.57 ± 0.0	2.58 ± 0.2	3.78 ± 0.1	3.02 ± 0.1
	OD+US_i_	2.68 ± 0.2	2.65 ± 0.1	3.14 ± 0.0	3.91 ± 0.2	3.96 ± 0.2
**150**	OD	2.18 ± 0.1	2.46 ± 0.1	2.72 ± 0.1	3.60 ± 0.0	2.93 ± 0.1
	OD+US	2.42 ± 0.2	2.55 ± 0.2	2.58 ± 0.2	3.87 ± 0.1	3.17 ± 0.2
	OD+US_i_	2.94 ± 0.3	2.40 ± 0.1	2.76 ± 0.1	4.10 ± 0.1	3.66 ± 0.2
**180**	OD	2.08 ± 0.1	2.54 ± 0.2	2.62 ± 0.1	3.38 ± 0.1	3.22 ± 0.2
	OD+US	1.79 ± 0.2	2.78 ± 0.2	2.53 ± 0.1	3.24 ± 0.0	3.05 ± 0.1
	OD+US_i_	2.15 ± 0.2	2.38 ± 0.0	2.87 ± 0.2	3.67 ± 0.2	3.15 ± 0.1

**Table 2:** The influence of osmotic agents and pre-treatment time on water content in fruit.

Factor		*p*-Value	Contrast	+/– Limits	Difference
Type of osmotic substance	erythritol ^b^	0.000 *	erythritol–xylitol	0.1300	–0.2106 *
	xylitol ^c^		erythritol–sucrose	0.1311	0.3703 *
	maltitol ^e^		xylitol–maltitol	0.1305	–0.7410 *
	DHA ^d^		xylitol–sucrose	0.1335	0.5809 *
	sucrose ^a^		DHA–erythritol	0.1385	0.6672 *
Time (min)	30 ^e^	0.000 *	30–60	0.1497	0.6378 *
	60 ^d^		60–90	0.1504	0.2674 *
	90 ^c^		90–120	0.1549	0.2734 *
	120 ^b^		120–150	0.1577	0.0955
	150 ^b^		120–180	0.1548	0.3013 *
	180 ^a^		150–180	0.1549	0.2059 *
Type of sonication	OD ^a^	0.000 *	OD–US	0.0902	–0.0117
	OD+US ^a^		OD–US_i_	0.0881	–0.3300 *
	OD+US_i_^b^		US–US_i_	0.0889	–0.3183 *

Statistical differences between factors; a Tukey test of main effects was performed. * Denotes a statistically significant difference. Means within columns with a different lowercase letter superscript are significantly different (*p* < 0.05).

**Table 3:** The influence of osmotic agents and pre-treatment time on water loss during OD.

Factor		*p*-Value	Contrast	+/– Limits	Difference
Type of osmotic substance	erythritol ^d^	0.000 *	erythritol–xylitol	0.0764	0.2053 *
	xylitol ^c^		erythritol–sucrose	0.0770	–0.2390 *
	maltitol ^a^		xylitol–maltitol	0.0767	0.5534 *
	DHA ^b^		xylitol–sucrose	0.0784	–0.4443 *
	sucrose ^e^		DHA–erythritol	0.0814	–0.2918 *
Time (min)	30 ^a^	0.000 *	30–60	0.0879	–0.2963 *
	60 ^b^		60–90	0.0888	–0.2109 *
	90 ^c^		90–120	0.0910	–0.1247 *
	120 ^d^		120–150	0.0927	–0.0552
	150 ^d^		120–180	0.0910	–0.1745 *
	180 ^e^		150–180	0.0910	–0.1193 *
Type of sonication	OD ^b^	0.000 *	OD–US	0.0530	–0.0034
	OD+US ^b^		OD–US_i_	0.0514	0.1406 *
	OD+US_i_^a^		US–US_i_	0.0522	0.1439 *

Statistical differences between factors; a Tukey test of main effects was performed. * Denotes a statistically significant difference. Means within columns with a different lowercase letter superscript are significantly different – homogeneous groups (*p* < 0.05).

**Table 4:** Values of *k*_1_, *k*_2_, R^2^, χ^2^, coefficient of residual variation (CRV), and RMSE of modelling WL using Peleg’s model. RMSE: root mean square error.

Solution	Application	*k_1_* (kg/kg·min)	*k_2_* (kg/kg)	R^2^	χ^2^	CRV (%)	RMSE
Erythritol	OD	16.714	0.534	0.862	0.006	5.85	0.064
	OD+US	25.757	0.424	0.982	0.000	1.59	0.018
	OD+US_i_	23.446	0.459	0.967	0.004	4.96	0.056
Xylitol	OD	21.117	0.558	0.942	0.004	5.26	0.054
	OD+US	43.499	0.431	0.959	0.004	5.94	0.005
	OD+US_i_	30.449	0.547	0.920	0.011	10.03	0.087
Maltitol	OD	45.191	0.846	0.910	0.000	3.18	0.019
	OD+US	35.390	1.210	0.906	0.001	5.63	0.028
	OD+US_i_	89.974	0.985	0.818	0.005	15.24	0.062
DHA	OD	13.019	0.658	0.976	0.002	3.51	0.034
	OD+US	34.449	0.512	0.884	0.016	11.89	0.106
	OD+US_i_	60.139	0.487	0.954	0.005	7.73	0.059
Sucrose	OD	28.202	0.362	0.974	0.005	4.88	0.060

**Table 5:** The influence of osmotic agents and pre-treatment time on solid gain during OD.

Factor		*p*-Value	Contrast	+/– Limits	Difference
Type of osmotic substance	erythritol ^b^	0.000 *	erythritol–xylitol	0.0479	0.0327
	xylitol ^b^		erythritol–sucrose	0.0483	–0.0906 *
	maltitol ^a^		xylitol–maltitol	0.0481	0.1408 *
	DHA ^a^		xylitol–sucrose	0.0492	–0.1233 *
	sucrose ^c^		DHA–erythritol	0.0511	–0.1875 *
Time (min)	30 ^a^	0.000 *	30–60	0.0552	–0.1396 *
	60 ^b^		60–90	0.0555	–0.3507
	90 ^b^		90–120	0.0571	–0.0801 *
	120 ^c^		120–150	0.0582	–0.0378
	150 ^cd^		120–180	0.0571	–0.0915 *
	180 ^d^		150–180	0.0571	–0.0538
Type of sonication	OD ^b^	0.000 *	OD–US	0.0333	0.0164
	OD+US ^b^		OD–US_i_	0.0325	0.0872 *
	OD+US_i_^a^		US–US_i_	0.0328	0.0708 *

Statistical differences between factors; a Tukey test of main effects was performed. * Denotes a statistically significant difference. Means within columns with a different lowercase letter superscript are significantly different – homogeneous groups (*p* < 0.05).

**Table 6:** Values of *k*_1_, *k*_2_, R^2^, χ^2^, CRV, and RMSE of modelling SG using Peleg’s model.

Solution	Application	*k_1_* (kg/kg·min)	*k_2_* (kg/kg)	R^2^	χ^2^	CRV (%)	RMSE
Erythritol	OD	103.136	0.782	0.906	0.002	10.65	0.041
	OD+US	66.307	1.302	0.699	0.009	22.11	0.079
	OD+US_i_	187.963	0.282	0.962	0.001	8.62	0.030
Xylitol	OD	89.979	1.108	0.899	0.002	9.88	0.034
	OD+US	114.672	0.806	0.869	0.006	17.48	0.065
	OD+US_i_	158.859	0.822	0.801	0.007	24.26	0.071
Maltitol	OD	138.805	1.398	0.849	0.004	23.47	0.052
	OD+US	7432.023	38.048	0.719	0.013	35.55	0.095
	OD+US_i_	186.212	3.007	0.484	0.004	25.93	0.053
DHA	OD	79.275	1.975	0.757	0.003	18.20	0.050
	OD+US	126.674	1.606	0.869	0.000	7.36	0.019
	OD+US_i_	1796.065	–7.29	0.854	0.001	22.53	0.029
Sucrose	OD	22.821	1.254	0.802	0.004	10.63	0.055

**Table 7:** The influence of osmotic agents and pre-treatment time on true water loss during OD.

Factor		*p*-Value	Contrast	+/– Limits	Difference
Type of osmotic substance	erythritol ^c^	0.000 *	erythritol–xylitol	0.0554	0.1245 *
	xylitol ^b^		erythritol–sucrose	0.0558	–0.1033 *
	maltitol ^a^		xylitol–maltitol	0.0556	0.3357 *
	DHA ^b^		xylitol–sucrose	0.0568	–0.2278 *
	sucrose ^d^		DHA–erythritol	0.0590	–0.0943 *
Time (min)	30 ^a^	0.000 *	30–60	0.0637	–0.1525 *
	60 ^b^		60–90	0.0640	–0.1351 *
	90 ^c^		90–120	0.0659	–0.0353
	120 ^cd^		120–150	0.0672	–0.0162
	150 ^cd^		120–180	0.0659	–0.0602
	180 ^d^		150–180	0.0660	–0.0440
Type of sonication	OD ^b^	0.000 *	OD–US	0.0384	–0.0106
	OD+US ^b^		OD–US_i_	0.0375	0.0563 *
	OD+US_i_^a^		US–US_i_	0.0379	0.0669 *

Statistical differences between factors; a Tukey test of main effects was performed. * Denotes a statistically significant difference. Means within columns with a different lowercase letter superscript are significantly different – homogeneous groups (*p* < 0.05).

**Table 8:** Values of *k*_1_, *k*_2_, R^2^, χ^2^, CRV, and RMSE of modelling WL_T_ using Peleg’s model.

Solution	Application	*k*_1_ (kg/kg·min)	*k*_2_ (kg/kg)	R^2^	χ^2^	CRV (%)	RMSE
Erythritol	OD	8.734	0.993	0.723	0.005	7.94	0.058
	OD+US	27.541	0.743	0.932	0.006	8.39	0.064
	OD+US_i_	10.986	0.900	0.934	0.003	5.74	0.044
Xylitol	OD	13.276	0.982	0.899	0.001	4.62	0.033
	OD+US	30.771	0.932	0.897	0.005	9.85	0.061
	OD+US_i_	24.939	0.947	0.817	0.012	15.01	0.095
Maltitol	OD	34.796	1.338	0.860	0.000	3.23	0.015
	OD+US	31.860	1.763	0.791	0.003	11.70	0.044
	OD+US_i_	95.468	1.349	0.836	0.003	14.91	0.047
DHA	OD	10.983	0.966	0.958	0.002	4.67	0.033
	OD+US	33.461	0.823	0.864	0.011	13.08	0.087
	OD+US_i_	34.938	0.818	0.955	0.003	6.91	0.045
Sucrose	OD	36.246	0.696	0.959	0.003	6.50	0.049

**Table 9:** The influence of osmotic agents and pre-treatment time on CR during OD.

Factor		*p*-Value	Contrast	+/– Limits	Difference
Type of osmotic substance	erythritol ^b^	0.000 *	erythritol–xylitol	0.0254	–0.0144
	xylitol ^b^		erythritol–sucrose	0.0256	0.0470 *
	maltitol ^c^		xylitol–maltitol	0.0255	–0.0721 *
	DHA ^c^		xylitol–sucrose	0.0260	0.0614 *
	sucrose ^a^		DHA–erythritol	0.0270	0.1009 *
Time (min)	30 ^d^	0.000 *	30–60	0.0292	0.0854 *
	60 ^c^		60–90	0.0293	0.0233
	90 ^c^		90–120	0.0302	0.0429 *
	120 ^b^		120–150	0.0308	0.0130
	150 ^ab^		120–180	0.0302	0.0415 *
	180 ^a^		150–180	0.0302	0.0285
Type of sonication	OD ^a^	0.000 *	OD–US	0.0176	–0.0034
	OD+US ^a^		OD–US_i_	0.0172	–0.0482 *
	OD+US_i_^b^		US–US_i_	0.0174	–0.0447 *

Statistical differences between factors; a Tukey test of main effects was performed. * Denotes a statistically significant difference. Means within columns with a different lowercase letter superscript are significantly different – homogeneous groups (*p* < 0.05).

**Table 10:** Values of *k*_1_, *k*_2_, R2, χ^2^, CRV, and RMSE of modelling CR using Peleg’s model.

Solution	Application	*k*_1_ (kg/kg·min)	*k_2_* (kg/kg)	R^2^	χ^2^	CRV (%)	RMSE
Erythritol	OD	139.433	1.645	0.959	0.001	3.28	0.019
	OD+US	85.026	2.295	0.794	0.002	5.92	0.036
	OD+US_i_	235.305	1.046	0.960	0.000	1.92	0.012
Xylitol	OD	124.570	1.997	0.942	0.000	3.23	0.020
	OD+US	137.717	1.700	0.889	0.001	4.75	0.028
	OD+US_i_	209.326	1.637	0.858	0.001	5.14	0.033
Maltitol	OD	255.096	2.046	0.877	0.002	5.05	0.034
	OD+US	94.274	3.031	0.664	0.002	5.75	0.037
	OD+US_i_	72.079	4.168	0.565	0.001	4.33	0.030
DHA	OD	77.326	3.007	0.893	0.001	4.15	0.027
	OD+US	138.372	2.578	0.903	0.000	1.97	0.013
	OD+US_i_	1479.120	–4.603	0.824	0.001	3.21	0.024
Sucrose	OD	23.758	2.260	0.967	0.001	3.83	0.020

**Table 11:** The influence of osmotic agents and pre-treatment time on water activity during OD.

Factor		*p*-Value	Contrast	+/– Limits	Difference
Type of osmotic substance	erythritol ^a^	0.000 *	erythritol–xylitol	0.0067	–0.0052
	xylitol ^ab^		erythritol–sucrose	0.0062	–0.0050
	maltitol ^c^		xylitol–maltitol	0.0062	–0.0246 *
	DHA ^b^		xylitol–sucrose	0.0063	0.0002
	sucrose ^ab^		DHA–erythritol	0.0066	0.0083 *
Time (min)	30 ^d^	0.000 *	30–60	0.0071	0.0080 *
	60 ^bc^		60–90	0.0071	–0.0035
	90 ^cd^		90–120	0.0074	0.0123 *
	120 ^a^		120–150	0.0075	–0.0037
	150 ^ab^		120–180	0.0074	–0.0009
	180 ^a^		150–180	0.0074	0.0028
Type of sonication	OD ^c^	0.000 *	OD–US	0.0043	0.0682 *
	OD+US ^a^		OD–US_i_	0.0042	0.0606 *
	OD+US_i_^b^		US–US_i_	0.0042	–0.0075 *

Statistical differences between factors; a Tukey test of main effects was performed. * Denotes a statistically significant difference. Means within columns with a different lowercase letter superscript are significantly different – homogeneous groups (*p* < 0.05).

**Table 12:** Values of the browning index (BI) parameter during OD in different solutions. OD: osmotic dehydration; US: continuous sonication; USi: interval sonication.

Time (min)	Solution/Application	Sucrose	Erythritol	Xylitol	Maltitol	DHA
**0**		20.67 ± 2.12				
**30**	OD	14.4 ± 1.3	16.6 ± 1.5	13.8 ± 0.2	14.3 ± 1.2	21.2 ± 4.5
	OD+US	25.3 ± 0.4	25.0 ± 0.5	31.2 ± 2.6	28.2 ± 1.4	24.3 ± 0.0
	OD+US_i_	27.6 ± 3.8	48.3 ± 0.9	39.6 ± 1.0	43.9 ± 3.0	30.0 ± 1.8
**60**	OD	17.6 ± 3.8	23.7 ± 0.7	24.6 ± 1.8	22.4 ± 3.0	22.0 ± 3.5
	OD+US	23.9 ± 1.7	26.8 ± 0.4	29.7 ± 0.6	31.1 ± 4.7	33.3 ± 3.0
	OD+US_i_	29.1 ± 1.6	48.2 ± 1.7	45.7 ± 0.0	41.8 ± 2.1	32.7 ± 1.7
**90**	OD	16.2 ± 3.1	22.1 ± 1.7	20.4 ± 4.2	20.9 ± 0.2	19.4 ± 2.9
	OD+US	24.9 ± 1.5	30.7 ± 2.8	28.9 ± 0.8	30.2 ± 0.8	28.7 ± 0.7
	OD+US_i_	29.5 ± 1.2	44.1 ± 0.5	29.1 ± 1.8	52.3 ± 5.7	36.6 ± 2.2
**120**	OD	16.9 ± 1.5	21.1 ± 1.2	19.4 ± 3.9	19.4 ± 2.6	23.1 ± 7.2
	OD+US	23.1 ± 0.2	27.7 ± 0.1	36.9 ± 1.5	26.7 ± 1.4	31.7 ± 0.1
	OD+US_i_	28.4 ± 0.0	45.5 ± 0.6	33.3 ± 1.6	46.8 ± 3.6	32.0 ± 1.0
**150**	OD	16.7 ± 2.8	19.7 ± 0.1	20.7 ± 4.8	21.5 ± 0.9	22.9 ± 2.0
	OD+US	24.3 ± 2.3	28.8 ± 0.9	32.3 ± 1.2	25.7 ± 0.1	24.5 ± 0.2
	OD+US_i_	28.4 ± 0.0	49.0 ± 5.3	30.7 ± 0.1	31.3 ± 0.4	43.3 ± 0.4
**180**	OD	15.0 ± 0.5	19.8 ± 1.7	19.8 ± 2.6	22.8 ± 1.5	22.4 ± 6.4
	OD+US	19.8 ± 0.1	26.5 ± 2.4	28.4 ± 2.5	33.8 ± 0.2	26.7 ± 0.2
	OD+US_i_	32.4 ± 1.5	48.0 ± 1.7	33.9 ± 0.3	33.5 ± 1.8	45.0 ± 1.0

**Table 13:** The influence of osmotic agents and pre-treatment time on the BI parameter during OD.

Factor		*p*-Value	Contrast	+/– Limits	Difference
Type of osmotic substance	erythritol ^c^	0.000 *	erythritol–xylitol	3.0435	3.1333 *
	xylitol ^b^		erythritol–sucrose	3.0435	8.4874 *
	maltitol ^bc^		xylitol–maltitol	3.0435	–1.6702
	DHA ^bc^		xylitol–sucrose	3.0435	5.3541 *
	sucrose ^a^		DHA–erythritol	3.0435	–2.9343
Time (min)	30 ^a^	0.130	30–60	3.4854	–3.2889
	60 ^a^		60–90	3.4854	1.3982
	90 ^a^		90–120	3.4854	–0.0402
	120 ^a^		120–150	3.4854	0.9655
	150 ^a^		120–180	3.4854	–0.2985
	180 ^a^		150–180	3.4854	–1.2640
Type of sonication	OD ^a^	0.000 *	OD–US	2.0206	–8.5294 *
	OD+US ^b^		OD–US_i_	2.0206	–18.3389 *
	OD+US_i_^c^		US–US_i_	2.0206	–9.8096 *

Statistical differences between factors; a Tukey test of main effects was performed. * Denotes a statistically significant difference. Means within columns with a different lowercase letter superscript are significantly different – homogeneous groups (*p* <0.05).

**Table 14:** Values of absolute colour difference (∆E) during OD in different solutions. OD: osmotic dehydration; US: continuous sonication; USi: interval sonication.

Time (min)	Solution/Application	Sucrose	Erythritol	Xylitol	Maltitol	DHA
**30**	OD	6.6 ± 1.1	5.7 ± 0.3	7.5 ± 0.3	7.0 ± 0.8	5.1 ± 1.3
	OD+US	5.1 ± 0.3	4.4 ± 0.1	8.6 ± 0.6	6.1 ± 0.5	4.4 ± 0.2
	OD+US_i_	5.9 ± 1.7	15.3 ± 0.1	12.5 ± 0.6	12.5 ± 1.0	6.4 ± 1.1
**60**	OD	6.2 ± 0.1	6.8 ± 0.7	7.4 ± 1.0	6.1 ± 0.8	5.9 ± 1.6
	OD+US	5.1 ± 1.0	7.4 ± 0.6	9.0 ± 0.7	8.9 ± 1.8	8.6 ± 1.4
	OD+US_i_	6.5 ± 0.9	16.2 ± 0.4	15.3 ± 0.2	15.3 ± 0.8	7.8 ± 0.9
**90**	OD	5.6 ± 0.6	7.2 ± 0.1	6.2 ± 0.7	5.5 ± 0.0	5.3 ± 2.4
	OD+US	5.0 ± 0.3	7.8 ± 1.4	8.5 ± 0.4	8.8 ± 0.4	7.5 ± 0.5
	OD+US_i_	7.0 ± 0.5	14.5 ± 0.0	7.9 ± 1.0	7.9 ± 1.8	10.4 ± 0.9
**120**	OD	5.2 ± 0.7	6.3 ± 0.7	6.7 ± 0.0	6.3 ± 0.8	6.2 ± 0.1
	OD+US	4.2 ± 0.0	7.5 ± 0.1	11.7 ± 0.3	6.8 ± 0.8	8.5 ± 0.2
	OD+US_i_	6.1 ± 0.6	15.9 ± 0.7	10.3 ± 0.8	10.3 ± 0.8	8.6 ± 0.2
**150**	OD	5.3 ± 0.4	6.3 ± 0.3	6.7 ± 0.5	4.8 ± 0.2	5.2 ± 0.1
	OD+US	4.3 ± 1.2	8.7 ± 0.0	10.4 ± 0.7	7.2 ± 0.3	6.4 ± 0.2
	OD+US_i_	6.7 ± 0.2	17.3 ± 1.3	9.0 ± 0.3	9.0 ± 0.0	14.5 ± 0.5
**180**	OD	5.7 ± 0.2	6.8 ± 0.4	6.1 ± 0.8	5.7 ± 0.4	5.9 ± 0.4
	OD+US	5.4 ± 0.4	6.5 ± 0.9	9.0 ± 1.1	10.2 ± 0.3	6.8 ± 0.9
	OD+US_i_	8.9 ± 0.5	15.8 ± 0.8	11.0 ± 0.0	11.0 ± 0.8	14.8 ± 0.4

**Table 15:** The influence of osmotic agents and pre-treatment time on absolute colour difference during OD.

Factor		P-Value	Contrast	+/– Limits	Difference
Type of osmotic substance	erythritol ^c^	0.000 *	erythritol–xylitol	1.4168	0.6972
	xylitol ^c^		erythritol–sucrose	1.4168	3.9778 *
	maltitol ^bc^		xylitol–maltitol	1.4168	0.1278
	DHA ^b^		xylitol–sucrose	1.4168	3.2806 *
	sucrose ^a^		DHA–erythritol	1.4168	–2.1333 *
Time (min)	30 ^a^	0.468	30–60	1.6225	–1.0933
	60 ^a^		60–90	1.6225	0.4500
	90 ^a^		90–120	1.6225	–0.0833
	120 ^a^		120–150	1.6225	0.2033
	150 ^a^		120–180	1.6225	–0.2233
	180 ^a^		150–180	1.6225	-0.4267
Type of sonication	OD ^a^	0.000 *	OD–US	0.9406	–1.1800 *
	OD+US ^b^		OD–US_i_	0.9406	–5.3083 *
	OD+US_i_^c^		US–US_i_	0.9406	–4.1283 *

Statistical differences between factors; a Tukey test of main effects was performed. * Denotes a statistically significant difference. Means within columns with a different lowercase letter superscript are significantly different – homogeneous groups (*p* < 0.05).

## Nomenclature

OD	osmotic dehydration
US	application of ultrasound
OD+US	ultrasound-assisted osmotic dehydration (continuous method)
OD+US_i_	ultrasound-assisted osmotic dehydration (interval method)
WC	water content, (g/g d.m.)
WL	water loss, (g/g i.d.m.)
WL_T_	true water loss (g/g d.m)
SG	solid gain, (g/g i.d.m.)
CR	Cichowska et al. ratio
*k_1_*	constant in Peleg’s model, (kg/kg)
*k_2_*	constant in Peleg’s model, (kg/kg·h)
*τ*	time of osmotic dehydration (min)
